# Interpreting Circulating Bile Acid Profiles in Pancreatic Cancer: The Role of Cholestasis and Its Management

**DOI:** 10.3390/cancers18152481

**Published:** 2026-08-02

**Authors:** Elisa Danese, Alessandro Esposito, Matteo De Pastena, Fabio Del Ben, Gabriella Lionetto, Alessia Scirpoli, Mariateresa Rizza, Roberto Salvia, Giuseppe Lippi

**Affiliations:** 1 Section of Clinical Biochemistry, Department of Engineering for Innovation Medicine, University of Verona, 37129 Verona, Italy; 2 Department of General and Pancreatic Surgery, Pancreas Institute, University of Verona Hospital Trust, 37129 Verona, Italy; 3 Immunopathology and Cancer Biomarkers Unit, Department of Cancer Research and Advanced Diagnostics, CRO Aviano National Cancer Institute IRCCS, 33081 Aviano, Italy

**Keywords:** bile acid, pancreatic ductal adenocarcinoma, UDCA, bilirubin, cholestasis

## Abstract

Background: Circulating bile acid (BA) profiles are increasingly explored in pancreatic cancer, although their interpretation is often complicated by biliary obstruction and its clinical management. In this study, we characterized plasma BA profiles in pancreatic ductal adenocarcinoma (PDAC) and assessed the relative contributions of tumor localization, histological subtype, cholestasis, and cholestasis-related interventions. Methods: Plasma BAs were quantified by LC-MS/MS in patients with PDAC of the pancreatic head (hPDAC, *n* = 132), PDAC of the body-tail (tPDAC, *n* = 42), and non-PDAC tumors of the pancreatic head (hnonPDAC, *n* = 34). BA concentrations and derived ratios were log-transformed and standardized, and their associations with bilirubin were examined using multivariable linear models, LOESS, and multivariate longitudinal analyses. Results: UDCA therapy was associated with markedly increased circulating UDCA, higher total BA concentrations, and enrichment of secondary BA species. Direct bilirubin explained more variability in BA composition than binary jaundice classification and showed a non-linear association with BA remodeling, with a distinct metabolic profile emerging only at high bilirubin levels. Longitudinally, BA profiles changed substantially over time in hPDAC, largely in parallel with bilirubin, whereas they remained comparatively stable in tPDAC. After adjustment for bilirubin, tumor-related differences were modest and context-dependent. Conclusions: Overall, circulating BA profiles in pancreatic cancer appear to be driven predominantly by cholestasis and its management rather than by tumor-related features alone.

## Simple Summary

Circulating bile acids are increasingly investigated as potential biomarkers in pancreatic cancer, but their composition may be substantially influenced by cholestasis and its clinical management. In this study, direct bilirubin, biliary drainage, and ursodeoxycholic acid treatment emerged as the principal determinants of circulating bile acid variability, whereas differences associated with tumor localization were more limited and context-dependent. Direct bilirubin provided greater explanatory value than binary jaundice classification and identified pronounced compositional remodeling at higher concentrations. These findings provide a clear framework for future biomarker studies and highlight the need to systematically account for bilirubin levels, biliary drainage history, and ursodeoxycholic acid exposure when interpreting circulating bile acid profiles.

## 1. Background

Pancreatic cancer (PC) is one of the most lethal malignancies [1], and currently represents the seventh leading cause of cancer-related death worldwide, with a 5-year survival rate of approximately 7%. Most cases are diagnosed at an advanced stage because of the lack of specific symptoms, contributing to the poor prognosis [2]. Pancreatic ductal adenocarcinoma (PDAC) accounts for approximately 90% of cases and frequently arises in the head of the pancreas, where tumor growth often leads to obstruction of the common bile duct [3]. Accordingly, a considerable rate of patients present with obstructive jaundice, hyperbilirubinemia, and altered circulating bile acid (BA) levels.

BAs are amphiphilic molecules synthesized from cholesterol in the liver and play a central role in lipid absorption and metabolic regulation. Through enterohepatic circulation and microbial transformation in the gut, primary BAs are converted into a range of secondary species, generating a complex and dynamic pool of metabolites with distinct physicochemical and biological properties [4].

In recent years, circulating BA profiles have attracted increasing interest in gastrointestinal cancers, including PC [5]. In tissues directly exposed to the bile, such as the liver, biliary tract, and gastrointestinal mucosa, BAs have been implicated in tumor-related processes through several mechanisms, including inflammatory signaling and mucin regulation [6,7,8,9]. In PC, however, the interpretation of BA alterations remains uncertain, although a possible link between BAs, microbiota, and PC biology has been hypothesized [10]. Experimental and clinical studies have reported heterogeneous and context-dependent findings, and it is still unclear to what extent circulating BA profiles reflect tumor-related biology rather than the clinical consequences of biliary obstruction [5,11]. This issue is particularly relevant in PDAC, because cholestasis is not only common, but is also frequently modified by clinical interventions. Patients with tumors in the pancreatic head often undergo biliary drainage before surgery, and many receive ursodeoxycholic acid (UDCA) as part of jaundice management. These interventions are not biologically neutral, as they may alter BA composition through different mechanisms, including pharmacological remodeling of the BA pool and mechanical relief of obstruction [12,13,14]. Patients with similar bilirubin values may nonetheless represent distinct metabolic states depending on the presence, severity, timing, and treatment of cholestasis. Failure to account for these factors may confound the interpretation of circulating BA profiles and contribute to the heterogeneity reported across studies.

Accordingly, before considering circulating BAs as potential markers of tumor-related processes in pancreatic cancer, the extent to which their variability is attributable to cholestasis and its management must be clearly delineated. We therefore aimed to characterize plasma BA profiles in pancreatic cancer and to assess whether the observed differences were primarily related to anatomical localization or also to histological subtype. To this end, we compared PDAC arising in the pancreatic head (hPDAC) and body-tail (tPDAC) with non-PDAC tumors of the pancreatic head (hnonPDAC) using integrated cross-sectional and longitudinal statistical approaches.

## 2. Results

### 2.1. Impact of UDCA Therapy on BA Profile in hPDAC

Because ursodeoxycholic acid (UDCA) is frequently prescribed to manage cholestatic symptoms, we first sought to establish how exogenous UDCA administration alters circulating bile acid (BA) composition in patients with hPDAC. We hypothesized that UDCA therapy significantly distorts both individual BA concentrations and overall pool composition. To test this hypothesis, we compared circulating BA profiles between hPDAC patients receiving UDCA therapy and those who were untreated. UDCA therapy was associated with marked changes in the circulating BA profile. Patients receiving UDCA showed a significant increase in circulating UDCA levels (median 2198.9 vs. 15.25 ng/mL; fold change 144.2; *p* < 0.001), together with higher levels of its conjugated derivatives GUDCA (fold change 71.3; *p* < 0.001) and TUDCA (fold change 40.0; *p* < 0.001).

Total BAs were increased in treated patients (fold change 2.98; *p* < 0.001). Secondary BAs were also elevated (fold change 7.03; *p* < 0.001), including both conjugated (fold change 6.39; *p* < 0.001) and unconjugated species (fold change 6.56; *p* < 0.001). Total conjugated BAs (fold change 2.42; *p* < 0.001) and unconjugated BAs (fold change 3.95; *p* < 0.001) were also both increased.

**Figure 1:**
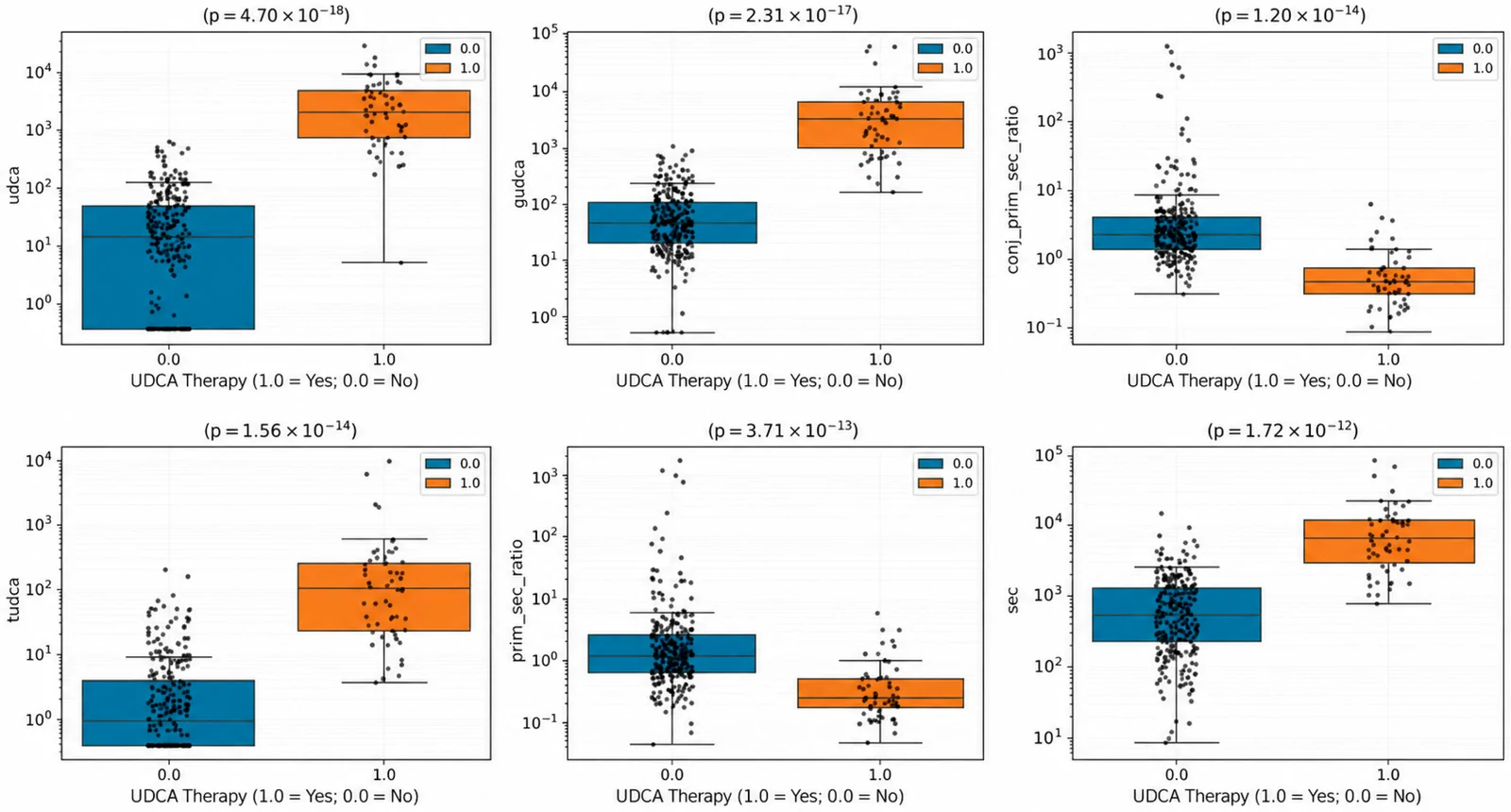
Effect of UDCA therapy on BA levels and composition in hPDAC. Boxplots of selected bile acids and derived ratios in patients with hPDAC with (*n* = 40) and without (*n* = 92) UDCA therapy. Values are shown on a log scale. Each point represents an individual subject.

Compositional ratios were significantly altered, with a reduction in the primary-to-secondary BA ratio (fold change 0.18; *p* < 0.001), as well as in the conjugated (fold change 0.20; *p* < 0.001) and unconjugated (fold change 0.29; *p* < 0.001) primary-to-secondary ratios (Figure 1). No significant differences were observed for several individual primary BAs, including CA and CDCA.

Given the effect of UDCA therapy on BA composition, all subsequent analyses were restricted to patients not receiving UDCA (*n* = 156). Clinical and pathological characteristics of this study population are summarized in Table 1.

### 2.2. Impact of Cholestasis on BA Profile in hPDAC

Biliary obstruction and clinical jaundice are hallmark features of pancreatic head tumors. However, it remains unclear whether categorical indicators (such as clinical jaundice or bilirubin above/below threshold) or continuous quantitative markers (such as direct bilirubin) capture systemic BA alterations more effectively. We hypothesized that quantitative direct bilirubin levels would serve as a superior continuous predictor of cholestasis-induced metabolic remodeling. To test this and determine the optimal predictor for downstream models, we first compared the explanatory power of binary clinical jaundice against quantitative direct bilirubin concentrations.

Direct bilirubin explained a greater proportion of variance (+4%, *p* = 0.002) and was therefore used as the primary predictor in subsequent analyses.

**Figure 2:**
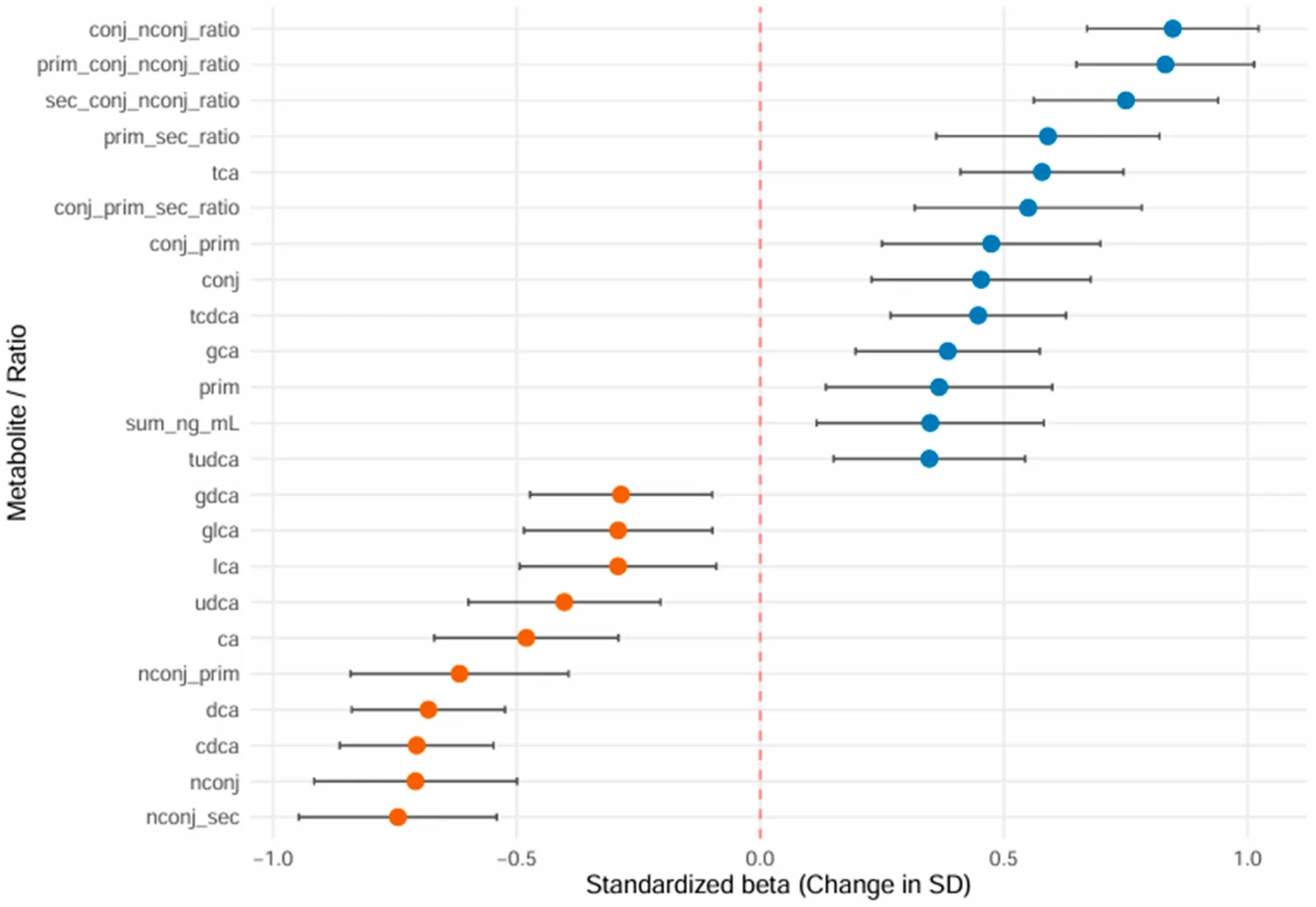
Association of direct bilirubin with circulating BA variables. Standardized β coefficients from linear models adjusted for age, sex, and cholesterol are shown with 95% confidence intervals. Each point represents the effect size of direct bilirubin on an individual BA or derived ratio. The vertical dashed line indicates no effect (β = 0). Blue points indicate positive associations and orange points negative associations.

When each BA parameter was tested within adjusted models including age, sex, and cholesterol, the conjugated-to-unconjugated BA ratio showed the strongest association, with the largest standardized effect size (β ≈ 0.9; *p* < 0.001) (Figure 2).

**Figure 3:**
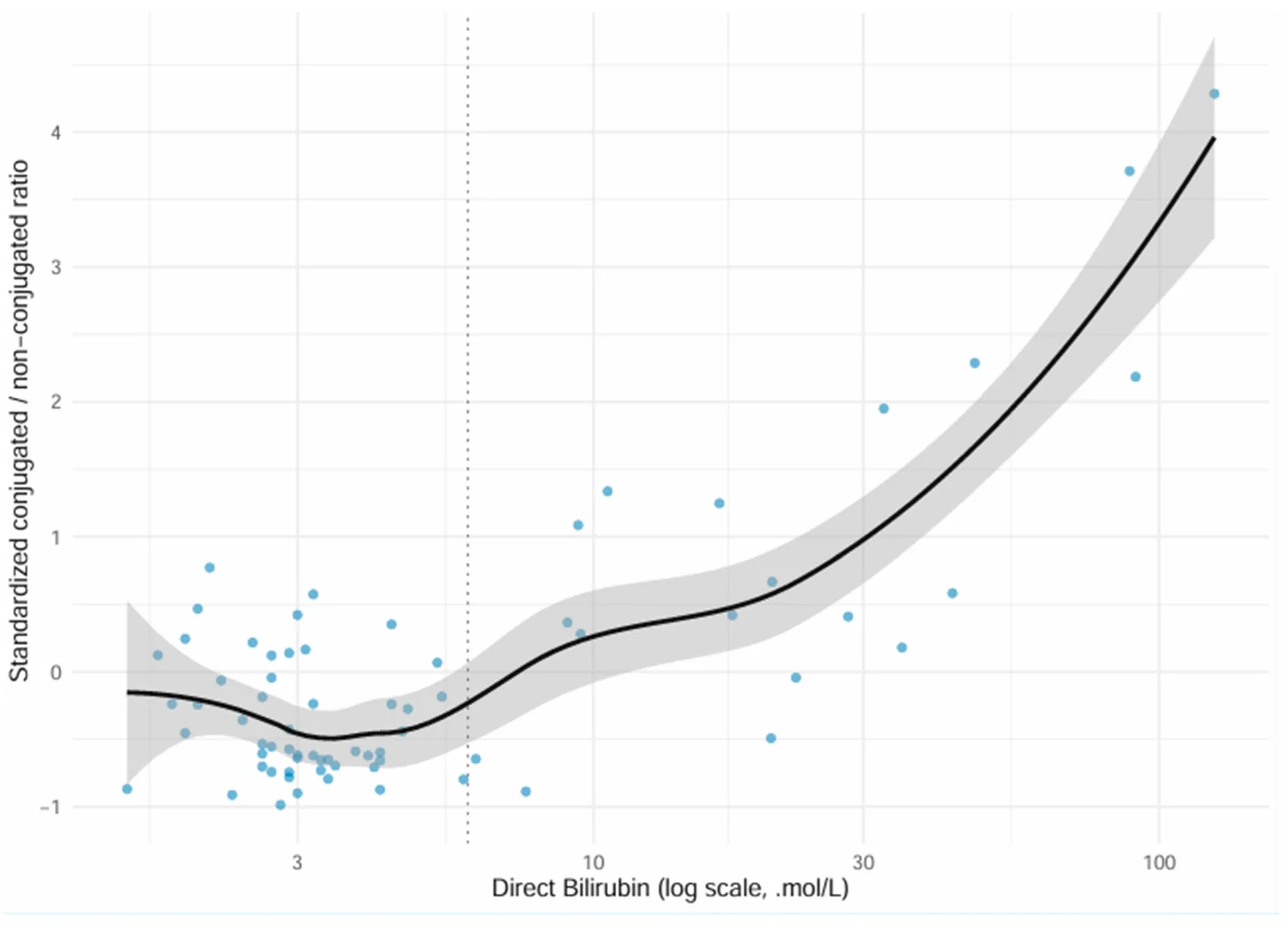
Relationship between direct bilirubin and the conjugated-to-unconjugated BA ratio. Scatter plot of direct bilirubin (log scale) versus the standardized conjugated-to-unconjugated BA ratio. The black line represents the LOESS fit and the shaded area the 95% confidence interval. The vertical dashed line marks 6 µmol/L. Three regions are apparent: a stable region below 6 µmol/L, a plateau between 6 and 30 µmol/L, and a sharp increase above 30 µmol/L.

Plotting this ratio against direct bilirubin revealed a non-linear relationship with three visually identifiable regions (Figure 3). Below 6 µmol/L, corresponding to the upper reference limit for direct bilirubin in our laboratory, ratio values remained relatively stable. Between 6 and approximately 30 µmol/L, a plateau was observed, whereas above approximately 30 µmol/L the ratio increased markedly. These empirically defined ranges were subsequently categorized as normal, moderate, and severe, respectively, to assess whether they were associated with differences in overall BA composition.

**Figure 4:**
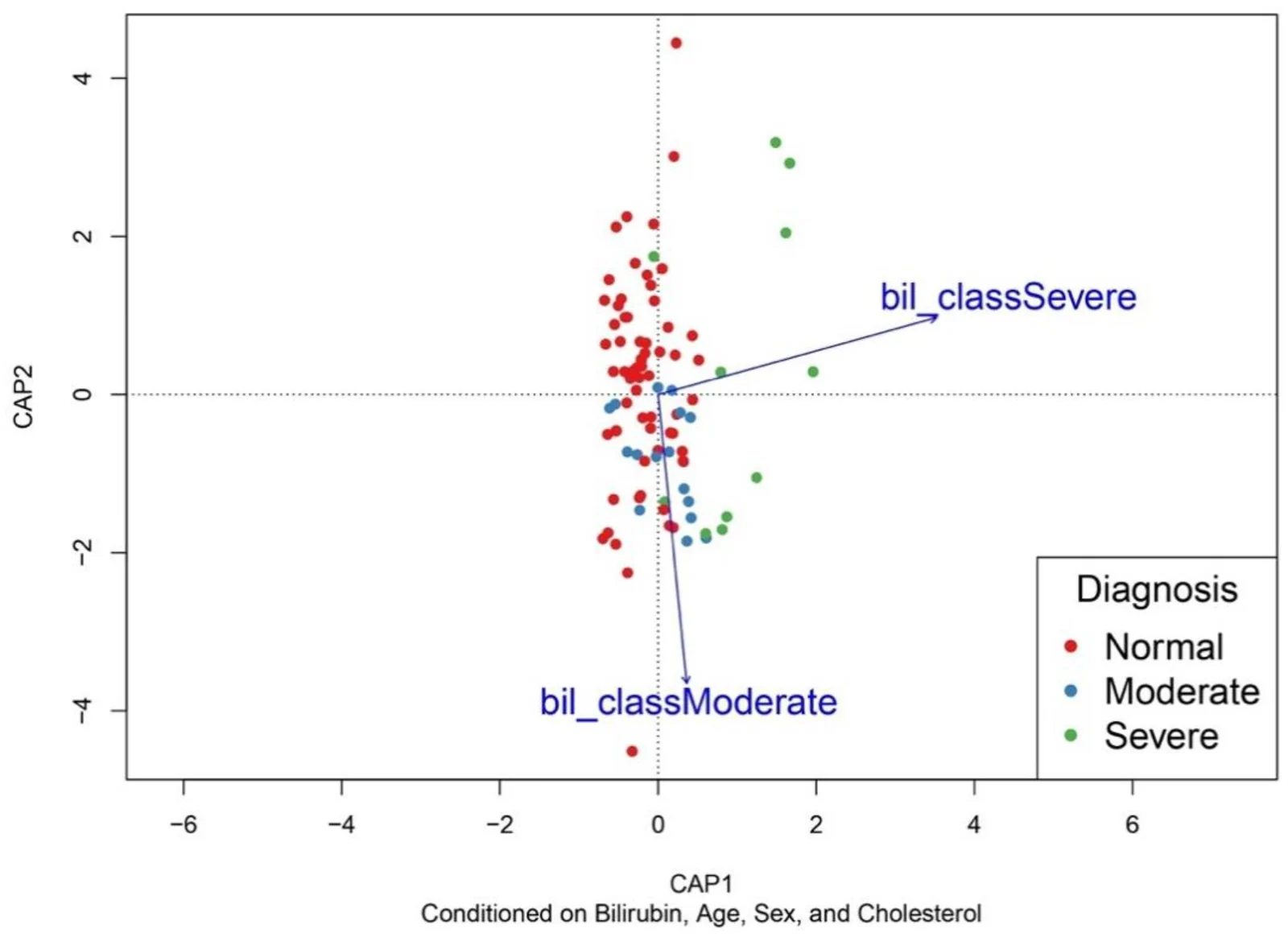
Distance-based redundancy analysis (dbRDA) of bile acid (BA) profiles across bilirubin severity classes. Ordination plot based on Bray–Curtis dissimilarity matrix of BA profiles, conditioned on potential confounders (age, sex, and total cholesterol). Individual points represent individual sample BA profiles color-coded by bilirubin class: Normal (red), Moderate (blue), and Severe (green). Constrained axes CAP1 and CAP2 display the variation in BA composition attributable to bilirubin severity class. Blue vectors indicate the direction and magnitude of multivariate shift for Moderate and Severe classes relative to the Normal baseline. Samples in the Severe group form a distinct cluster driven along CAP1, whereas Moderate samples show substantial overlap with the Normal bilirubin group.

To assess global metabolic variation, distance-based redundancy analysis (dbRDA) was performed using Bray–Curtis dissimilarity (Figure 4). The severe group formed a distinct cluster, whereas the moderate and normal groups showed substantial overlap. PERMANOVA showed that significant differences involved only the severe group, which differed from both the normal group (R^2^ = 0.22, *p* = 0.003) and the moderate group (R^2^ = 0.18, *p* = 0.006). No significant difference was observed between the moderate and normal groups (R^2^ = 0.03, *p* = 0.30).

### 2.3. Baseline Tumor-Related Differences After Stratification by Bilirubin Levels

**Figure 5:**
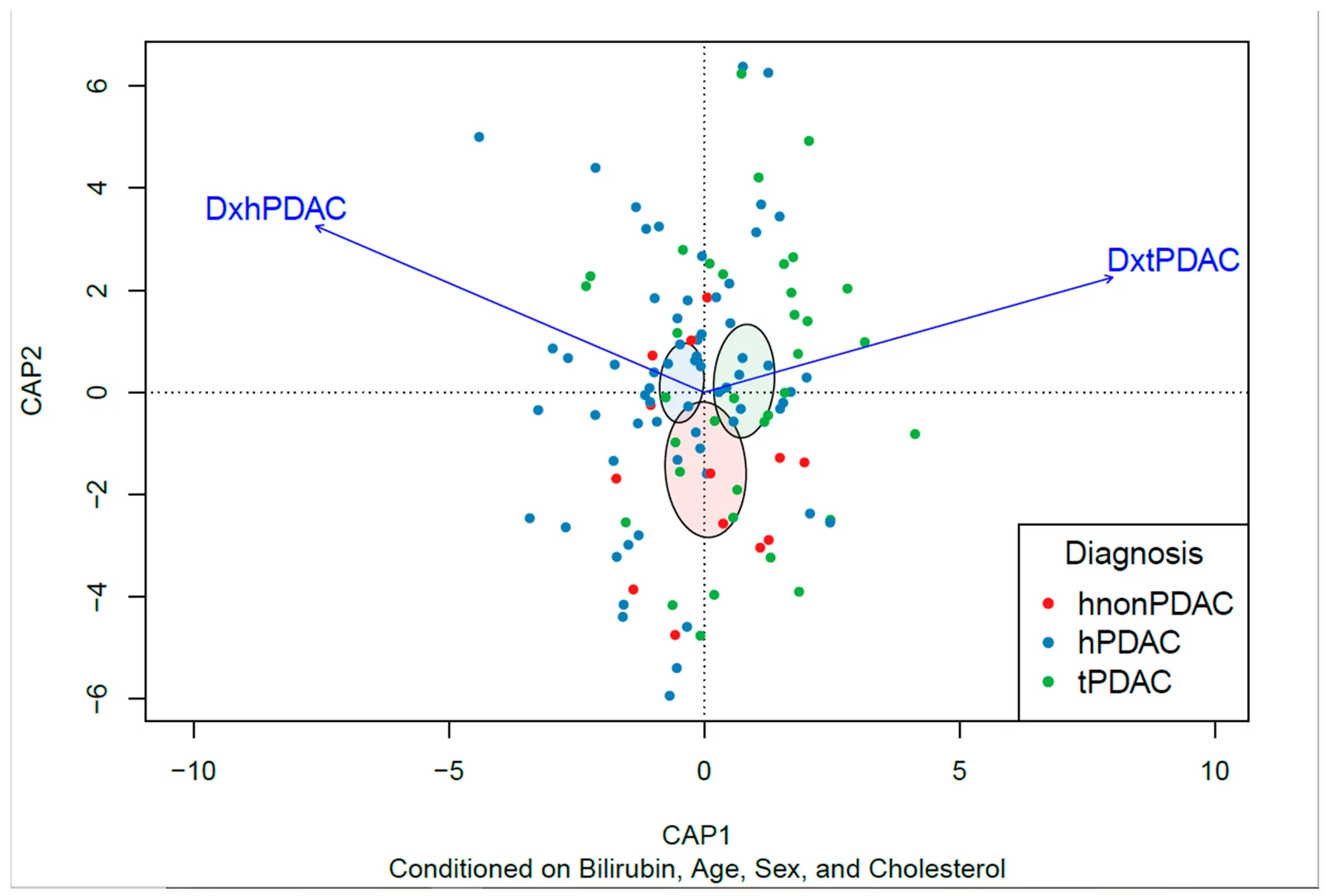
Canonical Analysis of Principal Coordinates (CAP) of baseline individual bile acid (BA) profiles across tumor groups. Ordination plot based on individual BA concentrations at baseline (T0), conditioned on potential confounders (bilirubin, age, sex, and total cholesterol). Individual subjects are represented by points color-coded by tumor classification: hnonPDAC (non-PDAC tumors of the pancreatic head; red), hPDAC (pancreatic head PDAC; blue), and tPDAC (pancreatic body/tail PDAC; green). Shaded ellipses denote 95% confidence intervals around group centroids. Blue vectors indicate the directional shift and magnitude of group centroids relative to the baseline reference group (hnonPDAC). Constrained axes CAP1 and CAP2 capture the multivariate variation attributable to tumor type. Partial separation along CAP1 is visible between tPDAC and head tumors, though substantial overlap remains across all three groups.

We next examined whether tumor diagnosis and anatomical location contributed to BA variability independently of current cholestatic burden. We hypothesized that tumor-related differences, if present, would be more detectable after stratification by direct bilirubin levels. To address this question, we conducted a stratified multivariate analysis across the three bilirubin classes, conditioning on relevant clinical covariates. Partial constrained analysis of principal coordinates (CAP), conditioned on bilirubin, age, sex, and cholesterol, revealed mild separation between tPDAC and tumors arising in the pancreatic head, with substantial overlap across groups (Figure 5). In PERMANOVA, tumor diagnosis explained a significant proportion of variance only within the normal bilirubin subgroup (R^2^ = 0.048, *p* = 0.005), but not in the moderate (*p* = 0.732) or severe (*p* = 0.455) groups. Within the normal subgroup, pairwise comparisons showed that tPDAC differed significantly from both hPDAC (*p* = 0.009) and hnonPDAC (*p* = 0.006), whereas no difference was observed between hPDAC and hnonPDAC (*p* = 1.000). Overall, baseline BA profiles in tPDAC were shifted toward unconjugated and secondary species, whereas tumors arising in the pancreatic head showed a profile more weighted toward conjugated BAs (Supplementary Figure S1).

### 2.4. Temporal Dynamics of BA Profiles in Operated Patients

**Figure 6:**
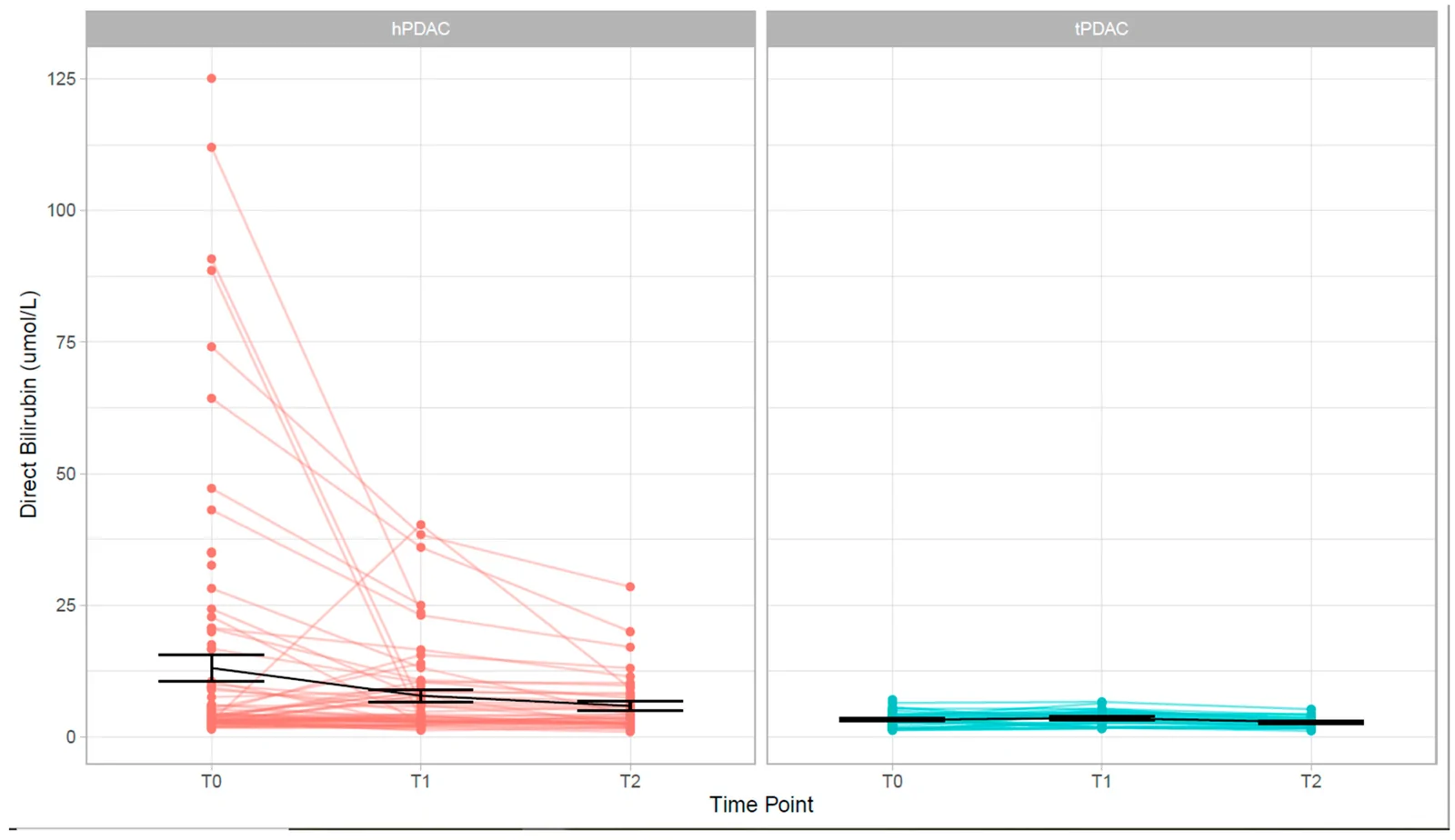
Temporal changes in direct bilirubin in operated patients with hPDAC and tPDAC. Direct bilirubin levels measured at preoperative baseline (T0), the first postoperative time point (T1), and hospital discharge (T2) in patients with hPDAC and tPDAC. A marked decline over time is observed in hPDAC, whereas tPDAC shows no comparable temporal trend.

To determine whether surgical resection and subsequent relief of biliary obstruction restore normal systemic BA profiles, we evaluated longitudinal metabolic trajectories in surgical patients. We hypothesized that surgical intervention in hPDAC patients would trigger a progressive normalization of BA composition in tandem with resolving cholestasis, whereas non-obstructive body/tail tumors (tPDAC) would maintain stable metabolic profiles over time. To test this hypothesis, we analyzed direct bilirubin and systemic BA dynamics preoperatively (T0) and across two postoperative time points (T1 and T2). Direct bilirubin levels showed a marked decline over time in hPDAC, whereas no comparable temporal trend was observed in tPDAC (Figure 6). Consistent with this pattern, BA profiles in hPDAC showed marked temporal variation. Multivariate analysis identified direct bilirubin as the main driver of variation (Bray–Curtis R^2^ = 0.061, *p* < 0.001; Euclidean R^2^ = 0.081, *p* < 0.001), with a significant effect of time (Bray–Curtis R^2^ = 0.013, *p* = 0.016; Euclidean R^2^ = 0.028, *p* = 0.001). Pairwise comparisons demonstrated significant differences between T0 and T1 (*p* = 0.007) and between T0 and T2 (*p* = 0.001), whereas no significant difference was observed between T1 and T2 (*p* = 0.221). In contrast, no significant temporal changes were observed in tPDAC: neither time (Bray–Curtis *p* = 0.136; Euclidean *p* = 0.091) nor direct bilirubin (Bray–Curtis *p* = 0.546; Euclidean *p* = 0.326) significantly contributed to variation in BA composition, and profiles remained stable across time points. Overall, these findings indicate that BA profiles in hPDAC are dynamic over time, whereas tPDAC profiles remain comparatively stable (Supplementary Figure S2). An additional comparison restricted to the later postoperative time point (T2 hPDAC vs. T2 tPDAC) still showed a significant association between diagnosis and BA composition after adjustment for age, sex, bilirubin, and cholesterol (Bray–Curtis R^2^ = 0.0446, *p* = 0.004; Euclidean R^2^ = 0.0385, *p* = 0.011).

## 3. Discussion

The present study shows that circulating BA profiles in PC are strongly influenced by cholestasis and its management, which emerged as major determinants of BA composition across cross-sectional, multivariate, and longitudinal analyses, whereas the contribution related to tumor localization was more limited and context-dependent.

A first major finding is the strong impact of UDCA therapy on the circulating BA profile in patients with head PDAC. This treatment was associated with a considerable increase in UDCA and its conjugated derivatives, together with an increase in the total BA pool and a shift in compositional ratios toward secondary species. These changes are consistent with the known pharmacokinetic properties of UDCA, including enterohepatic recirculation and extensive hepatic conjugation, which enrich the BA pool in more hydrophilic species and modify the relative abundance of endogenous BAs, as previously described in cholestatic liver disease [15,16,17,18,19,20].

To the best of our knowledge, this is the first study to characterize the overall impact of UDCA on circulating BA composition in a pancreatic cancer cohort, including changes extending beyond drug-derived metabolites to the distribution of broader BA classes.

This is relevant not only for PC but also for clinical settings characterized by obstructive jaundice, in which UDCA is often administered as part of clinical management, although its effect is rarely explicitly considered when interpreting circulating BA signatures.

A second major finding is the role of bilirubin as a quantitative determinant of BA composition. Compared with a binary classification of jaundice, direct bilirubin provided greater explanatory power and better captured the impact of cholestasis in our cohort. Its association with BA composition was non-linear, with limited variation at lower concentrations and a distinct metabolic profile emerging only at higher levels, whereas moderate elevations were not clearly distinguishable from normal values. This pattern is consistent with current concepts of cholestatic adaptation, whereby impaired biliary excretion promotes BA retention, remodeling of the circulating BA pool, and activation of compensatory regulatory pathways [12,20,21,22,23,24].

In pancreatic cancer, Liu et al. [25] similarly highlighted the influence of biliary obstruction on circulating BA profiles, but reported mainly an increase in total BA concentrations without significant changes in relative composition. The different findings may partly reflect our use of direct bilirubin as a continuous predictor across the full range of cholestatic burden, which allowed compositional changes at higher concentrations to become apparent. Differences in cohort characteristics, the number and types of BA species measured, analytical endpoints, and the timing and management of biliary obstruction may also have contributed. Overall, our results extend previous observations by indicating that marked cholestatic burden may affect both BA concentration and composition.

Regarding comparisons among tumor groups, differences in BA composition were overall limited and context-dependent. In baseline stratified analyses, tumor diagnosis explained only a small fraction of BA variability, and only in patients with normal bilirubin levels. Within this subgroup, differences emerged between tumors of the pancreatic tail and head, whereas no distinction was observed between PDAC and non-PDAC tumors located in the head. This result should be interpreted with caution, as patients with pancreatic head tumors and normal bilirubin had undergone biliary drainage before sampling. Therefore, the apparent association with anatomical localization may partly reflect the timing and metabolic consequences of jaundice resolution, not fully captured by bilirubin levels at T0.

Longitudinal analyses support this interpretation. In hPDAC, BA profiles changed markedly from preoperative to postoperative time points before stabilizing, whereas no significant temporal variation was observed in tPDAC. A modest difference between hPDAC and tPDAC persisted at the later postoperative time point. Overall, these findings suggest that baseline differences in patients with normal bilirubin may reflect residual effects of prior cholestasis and its treatment, although a limited contribution of anatomical localization cannot be excluded. Longer follow-up will be needed to clarify whether these differences persist or reflect transient post-intervention effects.

The compositional changes associated with cholestasis and UDCA exposure may also have biological relevance beyond their role as potential analytical confounders. Alterations in the balance between conjugated and unconjugated BAs, as well as between primary and secondary species, could influence signaling through BA-responsive receptors such as FXR and TGR5, with potential effects on metabolic regulation, inflammatory responses, epithelial function, and tumor–host interactions [23,24,26,27,28]. In pancreatic cancer, FXR overexpression has been associated with lymph-node metastasis, cell migration, and invasion, while broader evidence supports a potential role of BA signaling in pancreatic carcinogenesis [28,29]. These observations provide a mechanistic framework for interpreting the circulating BA alterations identified in our cohort and support further investigation of their biological relevance.

This study has some limitations. First, although the cohort was prospectively assembled at a single high-volume pancreatic surgery center and represents a substantial series of patients with resectable pancreatic and periampullary tumors, the single-center design may limit the generalizability of the findings. Some analytical subgroups, particularly the body-tail PDAC and periampullary non-PDAC groups, were smaller, reflecting the distribution of tumors encountered in surgical practice. In addition, several analyses were restricted to patients not receiving UDCA because of its marked effect on circulating BA composition, thereby reducing the number of patients available within specific analytical strata. This restriction reflects routine clinical management of biliary obstruction and was necessary to distinguish treatment-related effects from those associated with cholestasis and tumor characteristics. Second, although the statistical models accounted for the principal available clinical and biochemical covariates, residual confounding cannot be entirely excluded. UDCA exposure was specifically considered in the analyses, while corticosteroid and statin use were recorded and are reported in the Supplementary Table S1. Unmeasured variability in gut microbiota composition and intestinal transit may also have influenced primary-to-secondary BA relationships [26,27]. Third, the absence of healthy and non-malignant cholestatic control groups precludes assessment of the diagnostic specificity of the observed BA patterns.

The present findings should be interpreted primarily in the context of patients with pancreatic or periampullary tumors evaluated and managed in a surgical setting. Whether the same relationships between cholestatic burden, biliary management, and circulating BA profiles also apply to patients with unresectable disease or to those managed without surgery remains to be established. Nevertheless, the present results have broader methodological implications, as they indicate that direct bilirubin, biliary drainage history, and UDCA exposure are major determinants of circulating BA variability and should be systematically considered when interpreting BA profiles in future clinical and metabolomic studies.

## 4. Methods

### 4.1. Patient Selection

This prospective observational study included 208 consecutive patients enrolled between September 2022 and December 2023 at the General and Pancreatic Surgery Unit of the University Hospital of Verona. Patients who had undergone prior surgery were excluded, as were those with liver cirrhosis, acute liver failure, renal failure, multiple organ failure, or ongoing infection. Patients were stratified into three groups according to tumor type and anatomical localization: PDAC located in the head of the pancreas (hPDAC, *n* = 132), PDAC located in the body-tail of the pancreas (tPDAC, *n* = 42), and non-PDAC tumors arising in the head of the pancreas (hnonPDAC, *n* = 34). All cases were histopathologically confirmed. Clinical and pathological characteristics of the overall study population are reported in Supplementary Table S1.

For the cross-sectional analyses, plasma samples were collected in heparinized tubes prior to surgery (T0) and stored at −80 °C until analysis. All blood samples were collected at 08:00 a.m. after an overnight fast, to minimize variability related to food intake and diurnal variation. Before T0 sampling, all patients with tumors arising in the pancreatic head had received treatment for biliary obstruction, including UDCA therapy or biliary drainage. In a subset of operated patients with hPDAC and tPDAC, additional plasma samples were collected at the first postoperative time point (T1; hPDAC, *n* = 84 samples; tPDAC, *n* = 26 samples) and at hospital discharge (T2; hPDAC, *n* = 58 samples; tPDAC, *n* = 21 samples), and were then used for longitudinal analyses of BA profile dynamics. The study was approved by the Institutional Review Board of the University Hospital of Verona (3364CESC, 7 July 2021). All subjects provided written informed consent in accordance with institutional guidelines.

### 4.2. LC-MS/MS Analysis of BAs

Plasma BAs profiling was performed using a previously published and validated liquid chromatography–tandem mass spectrometry (LC–MS/MS) technique [30] with minor modifications. BA standards were obtained from Cambridge Isotope Laboratories (Andover, MA, USA) as commercially available mixtures of unconjugated (BA Standard Mix 1, MSK-BA1) and conjugated BAs (BA Standard Mix 2, MSK-BA2). These mixtures include primary and secondary BAs in both unconjugated and glycine- or taurine-conjugated forms (e.g., CA, CDCA, DCA, UDCA, LCA, and related conjugated species). Stable isotope-labeled BAs were provided as separate mixtures and used as internal standards. Plasma samples (200 μL) were subjected to methanol-based protein precipitation in the presence of internal standards, centrifuged, and the resulting supernatant was diluted prior to analysis. Reconstituted standards and processed samples were analyzed on a Cortecs T3 column using an ACQUITY UPLC I-Class system coupled to a Xevo TQ-S micro MS/MS operating in negative electrospray ionization and multiple reaction monitoring (MRM) mode. Detailed chromatographic and mass spectrometric conditions, together with analytical performance characteristics, have been previously described [30].

### 4.3. Statistical Analysis

Continuous variables were expressed as medians and interquartile ranges (IQRs). To account for non-normal distribution and high dynamic range, BA concentrations and derived ratios were log-transformed and standardized using Z-score scaling before analysis. Differences between independent groups were assessed using the Mann–Whitney U test, with Benjamini–Hochberg correction for multiple testing. Associations between clinical variables and BA profiles were evaluated using multivariable linear regression models, and standardized beta coefficients with 95% confidence intervals were calculated. Non-linear relationships between direct bilirubin and BA variables were explored using locally estimated scatterplot smoothing (LOESS), and variance partitioning was applied to compare the explanatory power of direct bilirubin and binary jaundice status. Global metabolic variation was assessed using PERMANOVA and visualized by constrained ordination methods, including dbRDA and partial constrained analysis of principal coordinates (CAP), after adjustment for relevant confounders. In addition, principal component analysis (PCA) of BA ratios was used as an exploratory approach to aid interpretation of group-related compositional shifts. Temporal changes in BA composition were assessed using PERMANOVA models, with pairwise comparisons performed when appropriate. Statistical analyses were performed using R v4.5.2, adonis, factoextra v.2.0.0, FactoMineR v2.13, pairwiseAdonis v0.4.1, vegan v2.7.3.

## 5. Conclusions

In conclusion, circulating BA profiles in pancreatic cancer were primarily shaped by cholestatic burden and its clinical management, particularly UDCA exposure and prior biliary intervention. Direct bilirubin was more informative than binary jaundice classification and captured a non-linear remodeling of BA composition that became apparent at higher concentrations, whereas tumor-related differences were modest and context-dependent. Future metabolomic and biomarker studies should routinely incorporate direct bilirubin, preferably as a continuous covariate, together with biliary drainage history and UDCA exposure before interpreting BA alterations as tumor-related. Prospective multicenter studies are required to validate these observations and to determine whether BA profiling adjusted for cholestatic and treatment-related factors provides independent diagnostic or prognostic information.

## Figures and Tables

**Table 1:** Clinical and pathological characteristics of the study population after exclusion of patients receiving UDCA therapy.

Characteristic	tPDAC (*n* = 37)	hPDAC (*n* = 92)	hnonPDAC (*n* = 27)
**Age (years)**, Median (IQR)	67 (12)	68 (14)	66 (16)
**Sex (Male)**, *n* (%)	16 (43.2%)	45 (48.9%)	17 (63.0%)
**BMI (kg/m^2^)**, Median (IQR)	25.7 (5.4)	24.2 (3.5)	23.1 (4.1)
**Jaundice (Present)**, *n* (%)	2 (5.4%)	28 (30.4%)	14 (51.9%)
**Direct Bilirubin (µmol/L)**, Median (IQR)	3.0 (1.8)	3.5 (6.7)	8.0 (11.3)
**Total Cholesterol (mmol/L)**, Median (IQR)	5.0 (1.5)	4.7 (1.7)	4.7 (1.8)

## Data Availability

Data are available on request to the authors.

## Supplementary Materials

The following supporting information can be downloaded at: https://www.mdpi.com/article/10.3390/cancers18152481/s1, Figure S1: PCA loading plot of bile acid ratios. Arrows represent the contribution of bile acid-derived ratios to the first two principal components. Ratios associated with secondary and unconjugated bile acids align with positive Dim1 values, consistent with the tPDAC profile, whereas conjugation-related ratios orient in the opposite direction, consistent with tumors arising in the pancreatic head. Arrow length and color indicate the relative contribution of each variable. Figure S2: Adjusted longitudinal evolution of the bile acid profile (PC1) in hPDAC and tPDAC. Arrows represent the contribution of bile acid-derived ratios to the first two principal components. Ratios associated with secondary and unconjugated bile acids align with positive Dim1 values, consistent with the tPDAC profile, whereas conjugation-related ratios orient in the opposite direction, consistent with tumors arising in the pancreatic head. Arrow length and color indicate the relative contribution of each variable. Table S1: Clinical and pathological characteristics of the overall study population.

## Abbreviations

Tauroursodeoxycholic acid (TUDCA), taurocholic acid (TCA), glycoursodeoxycholic acid (GUDCA), glycocholic acid (GCA), taurochenodeoxycholic acid (TCDCA), taurodeoxy-cholic acid (TDCA), cholic acid (CA), ursodeoxycholic acid (UDCA), glycochenodeoxy-cholic acid (GCDCA), glycodeoxycholic acid (GDCA), lithocholic acid (LCA), tauro-lithocholic acid (TLCA), chenodeoxycholic acid (CDCA), glycolithocholic acid (GLCA), deoxycholic acid (DCA), conjugated (conj), and unconjugated (unconj).

## References

[1] Lippi G., Mattiuzzi C. (2020). The Global Burden of Pancreatic Cancer. Arch. Med. Sci..

[2] Sung H., Ferlay J., Siegel R.L., Laversanne M., Soerjomataram I., Jemal A., Bray F. (2021). Global Cancer Statistics 2020: GLOBOCAN Estimates of Incidence and Mortality Worldwide for 36 Cancers in 185 Countries. CA A Cancer J. Clin..

[3] Luo G., Fan Z., Gong Y., Jin K., Yang C., Cheng H., Huang D., Ni Q., Liu C., Yu X. (2019). Characteristics and Outcomes of Pancreatic Cancer by Histological Subtypes. Pancreas.

[4] Chen M., Liu C., Wan Y., Yang L., Jiang S., Qian D., Duan J. (2021). Enterohepatic Circulation of Bile Acids and Their Emerging Roles on Glucolipid Metabolism. Steroids.

[5] Wang Y., Xu H., Zhang X., Ma J., Xue S., Shentu D., Mao T., Li S., Yue M., Cui J. (2024). The Role of Bile Acids in Pancreatic Cancer. Curr. Cancer Drug Targets.

[6] Danese E., Ruzzenente A., Montagnana M., Lievens P.M.-J. (2018). Current and Future Roles of Mucins in Cholangiocarcinoma—Recent Evidences for a Possible Interplay with Bile Acids. Ann. Transl. Med..

[7] Jia W., Xie G., Jia W. (2018). Bile Acid–Microbiota Crosstalk in Gastrointestinal Inflammation and Carcinogenesis. Nat. Rev. Gastroenterol. Hepatol..

[8] Di Ciaula A., Wang D.Q.-H., Molina-Molina E., Lunardi Baccetto R., Calamita G., Palmieri V.O., Portincasa P. (2017). Bile Acids and Cancer: Direct and Environmental-Dependent Effects. Ann. Hepatol..

[9] Danese E., Lievens P.M.-J., Padoan A., Peserico D., Galavotti R., Negrini D., Gelati M., Conci S., Ruzzenente A., Salvagno G.L. (2023). Plasma Bile Acid Profiling and Modulation of Secreted Mucin 5AC in Cholangiocarcinoma. Int. J. Mol. Sci..

[10] Malhotra P., Palanisamy R., Caparros-Martin J.A., Falasca M. (2023). Bile Acids and Microbiota Interplay in Pancreatic Cancer. Cancers.

[11] Režen T., Rozman D., Kovács T., Kovács P., Sipos A., Bai P., Mikó E. (2022). The Role of Bile Acids in Carcinogenesis. Cell. Mol. Life Sci..

[12] Kosuge T., Beppu T., Iwasaki S., Itoh T., Idezuki Y. (1990). Bile Acid Profile and Decrement Rate of Serum Total Bilirubin after Biliary Drainage. Gastroenterol. Jpn..

[13] Kovács P., Schwarcz S., Nyerges P., Bíró T.I., Ujlaki G., Bai P., Mikó E. (2024). Anticarcinogenic Effects of Ursodeoxycholic Acid in Pancreatic Adenocarcinoma Cell Models. Front. Cell Dev. Biol..

[14] McNally B.B., Carey E.J. (2025). Cholestatic Liver Diseases: Modern Therapeutics. Expert. Rev. Gastroenterol. Hepatol..

[15] Batta A.K., Arora R., Salen G., Tint G.S., Eskreis D., Katz S. (1989). Characterization of Serum and Urinary Bile Acids in Patients with Primary Biliary Cirrhosis by Gas-Liquid Chromatography-Mass Spectrometry: Effect of Ursodeoxycholic Acid Treatment. J. Lipid Res..

[16] Lazaridis K.N., Gores G.J., Lindor K.D. (2001). Ursodeoxycholic Acid ‘Mechanisms of Action and Clinical Use in Hepatobiliary Disorders’. J. Hepatol..

[17] Paumgartner G., Beuers U. (2004). Mechanisms of Action and Therapeutic Efficacy of Ursodeoxycholic Acid in Cholestatic Liver Disease. Clin. Liver Dis..

[18] Stiehl A., Raedsch R., Rudolph G., Walker S. (1984). Effect of Ursodeoxycholic Acid on Biliary Bile Acid and Bile Lipid Composition in Gallstone Patients. Hepatology.

[19] Mazzella G., Parini P., Bazzoli F., Villanova N., Festi D., Aldini R., Roda A., Cipolla A., Polimeni C., Tonelli D. (1993). Ursodeoxycholic Acid Administration on Bile Acid Metabolism in Patients with Early Stages of Primary Biliary Cirrhosis. Dig. Dis. Sci..

[20] Cabrera D., Arab J.P., Arrese M., Fiorucci S., Distrutti E. (2019). UDCA, NorUDCA, and TUDCA in Liver Diseases: A Review of Their Mechanisms of Action and Clinical Applications. Bile Acids and Their Receptors.

[21] Fuchs C.D., Simbrunner B., Baumgartner M., Campbell C., Reiberger T., Trauner M. (2025). Bile Acid Metabolism and Signalling in Liver Disease. J. Hepatol..

[22] Mousa O.Y., Juran B.D., McCauley B.M., Vesterhus M.N., Folseraas T., Turgeon C.T., Ali A.H., Schlicht E.M., Atkinson E.J., Hu C. (2021). Bile Acid Profiles in Primary Sclerosing Cholangitis and Their Ability to Predict Hepatic Decompensation. Hepatology.

[23] Perino A., Demagny H., Velazquez-Villegas L., Schoonjans K. (2021). Molecular Physiology of Bile Acid Signaling in Health, Disease, and Aging. Physiol. Rev..

[24] Chiang J.Y.L., Ferrell J.M. (2020). Bile Acid Receptors FXR and TGR5 Signaling in Fatty Liver Diseases and Therapy. Am. J. Physiol. Gastrointest. Liver Physiol..

[25] Liu C., Qin H., Wu Z., Liu J., Wang M., Yao J., Shang D., Yin P. (2026). Prognostic and Diagnostic Value of Serum Bile Acid Profiles in Pancreatic Cancer: A Retrospective Multicenter Cohort Study. J. Gastrointest. Cancer.

[26] Collins S.L., Stine J.G., Bisanz J.E., Okafor C.D., Patterson A.D. (2023). Bile Acids and the Gut Microbiota: Metabolic Interactions and Impacts on Disease. Nat. Rev. Microbiol..

[27] Cai J., Sun L., Gonzalez F.J. (2022). Gut Microbiota-Derived Bile Acids in Intestinal Immunity, Inflammation, and Tumorigenesis. Cell Host Microbe.

[28] Sharma B., Twelker K., Nguyen C., Ellis S., Bhatia N.D., Kuschner Z., Agriantonis A., Agriantonis G., Arnold M., Dave J. (2024). Bile Acids in Pancreatic Carcinogenesis. Metabolites.

[29] Lee J.Y., Lee K.T., Lee J.K., Lee K.H., Jang K.-T., Heo J.S., Choi S.H., Kim Y., Rhee J.C. (2011). Farnesoid X Receptor, Overexpressed in Pancreatic Cancer with Lymph Node Metastasis Promotes Cell Migration and Invasion. Br. J. Cancer.

[30] Danese E., Negrini D., Pucci M., De Nitto S., Ambrogi D., Donzelli S., Lievens P.M.-J., Salvagno G.L., Lippi G. (2020). Bile Acids Quantification by Liquid Chromatography–Tandem Mass Spectrometry: Method Validation, Reference Range, and Interference Study. Diagnostics.

