# Correction: Danese et al. Interpreting Circulating Bile Acid Profiles in Pancreatic Cancer: The Role of Cholestasis and Its Management. *Cancers* 2026, *18*, 2481

**DOI:** 10.3390/cancers18193144

**Published:** 2026-09-29

**Authors:** Elisa Danese, Alessandro Esposito, Matteo De Pastena, Fabio Del Ben, Gabriella Lionetto, Alessia Scirpoli, Mariateresa Rizza, Roberto Salvia, Giuseppe Lippi

**Affiliations:** 1 Section of Clinical Biochemistry, Department of Engineering for Innovation Medicine, University of Verona, 37129 Verona, Italy; mariateresa.rizza@studenti.univr.it (M.R.); giuseppe.lippi@univr.it (G.L.); 2 Department of General and Pancreatic Surgery, Pancreas Institute, University of Verona Hospital Trust, 37129 Verona, Italy; alessandro.esposito@aovr.veneto.it (A.E.); matteo.depastena@aovr.veneto.it (M.D.P.); gabriella.lionetto@univr.it (G.L.); alessiascirpoli@gmail.com (A.S.); roberto.salvia@univr.it (R.S.); 3 Immunopathology and Cancer Biomarkers Unit, Department of Cancer Research and Advanced Diagnostics, CRO Aviano National Cancer Institute IRCCS, 33081 Aviano, Italy

## Error in Figure

In the original publication [1], there was a mistake in Figure 4 as published. The image displayed as Figure 4 was incorrect and duplicated the image used for Figure 5, although the legend for Figure 4 remained correct. The corrected Figure 4 appears below. The authors state that the scientific conclusions are unaffected. This correction was approved by the Academic Editor. The original publication has also been updated.

## Figures and Tables

**Figure 4:**
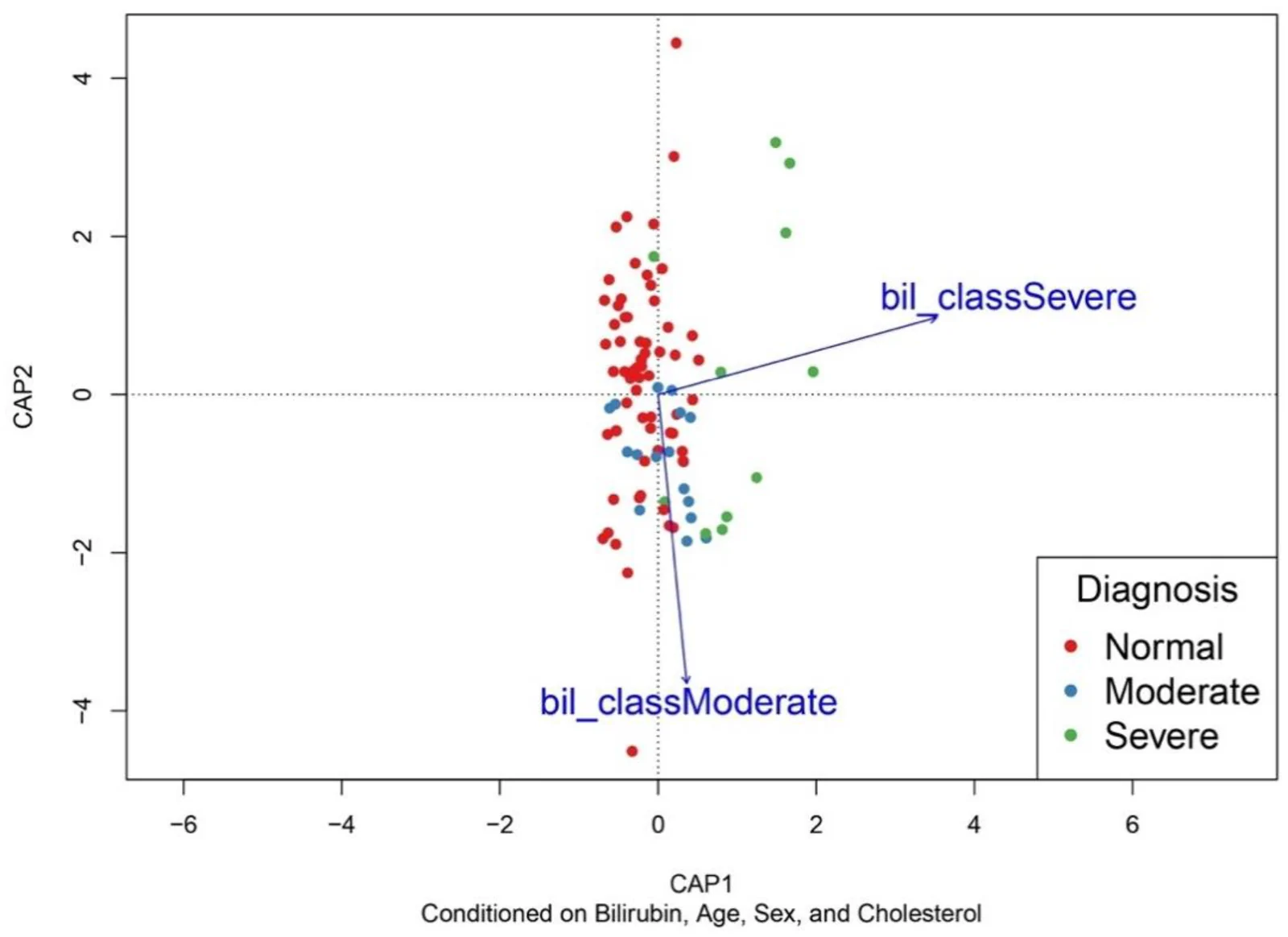
Distance-based redundancy analysis (dbRDA) of bile acid (BA) profiles across bilirubin severity classes. Ordination plot based on Bray–Curtis dissimilarity matrix of BA profiles, conditioned on potential confounders (age, sex, and total cholesterol). Individual points represent individual sample BA profiles color-coded by bilirubin class: Normal (red), Moderate (blue), and Severe (green). Constrained axes CAP1 and CAP2 display the variation in BA composition attributable to bilirubin severity class. Blue vectors indicate the direction and magnitude of multivariate shift for Moderate and Severe classes relative to the Normal baseline. Samples in the Severe group form a distinct cluster driven along CAP1, whereas Moderate samples show substantial overlap with the Normal bilirubin group.

## References

[1] Danese E., Esposito A., De Pastena M., Del Ben F., Lionetto G., Scirpoli A., Rizza M., Salvia R., Lippi G. (2026). Interpreting Circulating Bile Acid Profiles in Pancreatic Cancer: The Role of Cholestasis and Its Management. Cancers.

