# Supplementary material for: Interpreting Circulating Bile Acid Profiles in Pancreatic Cancer: The Role of Cholestasis and Its Management
Source: Cancers (Basel). 2026 Aug 2;18(15):2481. doi: 10.3390/cancers18152481 (PMC13463877; doi:10.3390/cancers18152481)
Supplement: Supplementary file 1 [file cancers-18-02481-s001.zip › cancers-4396027-supplementary-author revised.pdf]

Clinical and pathological characteristics of the overall study population

Supplementary table S1

**Characteristic**	**Available data, *n***	**hPDAC (*N*=132)**
**Age (years)**, *Median (Q1– Q3)*	132	67.0 (58.8–72.3)
**Sex (Male)**, *n (%)*	132	64 (48.5%)
**BMI (kg/m²)**, *Median (Q1–Q3)*	130	24.3 (22.1–26.1)
**Malignant,** *n (%)*	132	132 (100.0%)
**Total Bilirubin (µmol/L)** *Median (Q1–Q3)*	132	8.4 (5.8–16.4)
**Direct Bilirubin (µmol/L)** *Median (Q1–Q3)*	132	3.8 (2.7–10.5)
**UDCA,** *n (%)*	132	40 (30.3%)
**Steroids,** *n (%)*	128	9 (7.0%)
**Statins,** n (%)	130	27 (20.8%)
**Autoimmunity,** *n (%)*	128	8 (6.3%)
**Previous Cholangitis,** *n (%)*	129	18 (14.0%)
**NAT,** *n (%)*	132	79 (59.8%)
**CA 19.9 (kU/L),** *Median (Q1–Q3)*	130	64.5 (18.5–191.0)

**Characteristic**	**Available data, *n***	**tPDAC (*N*=42)**
**Age (years)**, *Median (Q1-Q3)*	42	67.5 (62.5–73.0)
**Sex (Male)**, *n (%)*	42	18 (42.9%)
**BMI (kg/m²)**, *Median (Q1-Q3)*	41	25.1 (22.0–27.4)
**Malignant,** *n (%)*	42	42 (100.0%)
**Total Bilirubin (µmol/L)** *Median (Q1-Q3)*	42	7.4 (5.1–11.0)
**Direct Bilirubin (µmol/L)** *Median (Q1-Q3)*	42	3.0 (2.2–4.2)
**UDCA,** *n (%)*	42	5 (11.9%)
**Steroids,** *n (%)*	40	0 (0.0%)
**Statins,** n (%)	40	5 (12.5%)
**Autoimmunity,** *n (%)*	40	5 (12.5%)
**Previous Cholangitis,** *n (%)*	41	0 (0.0%)
**NAT,** *n (%)*	42	21 (50.0%)
**CA 19.9 (kU/L),** *Median (Q1-Q3)*	42	53.5 (17.3–159.0)

**Characteristic**		**Available data, *n***	**hnon-PDAC (*N*=34)**
**Age (years)**, *Median (Q1–Q3)*		34	67.5 (61.3–73.8)
**Sex (Male)**, *n (%)*		34	23 (67.6%)
**BMI (kg/m²)**, *Median (Q1–Q3)*		34	23.3 (21.7–25.9)
**Malignant,** *n (%)*		34	34 (100.0%)
**Total Bilirubin (µmol/L)** *Median (Q1–Q3)*		34	11.0 (6.3–21.4)
**Direct Bilirubin (µmol/L)** *Median (Q1–Q3)*		34	6.5 (3.5–14.8)
**UDCA,** *n (%)*		34	7 (20.6%)
**Steroids,** *n (%)*		32	2 (6.3%)
**Statins,** n (%)		30	8 (26.7%)
**Autoimmunity,** *n (%)*		33	5 (15.2%)
**Previous Cholangitis,** *n (%)*		33	4 (12.1%)
**NAT,** *n (%)*		34	3 (8.8%)
**CA 19.9 (kU/L),** *Median (Q1–Q3)*		33	15.0 (9.0–41.0)
**Histological subtype,** *n (%)*	**AAC** (Ampullary Adenocarcinoma)	34	23 (67.6%)
	**DAC** (Ductal Adenocarcinoma)	34	6 (17.6%)
	**DBD** (Duodenobiliary Differentiated Adenocarcinoma)	34	5 (14.7%)
