# Supplementary material for: Interpreting Circulating Bile Acid Profiles in Pancreatic Cancer: The Role of Cholestasis and Its Management
Source: Cancers (Basel). 2026 Aug 2;18(15):2481. doi: 10.3390/cancers18152481 (PMC13463877; doi:10.3390/cancers18152481)
Supplement: Supplementary file 1 [file cancers-18-02481-s001.zip › Supplementary Figure S1.pdf]

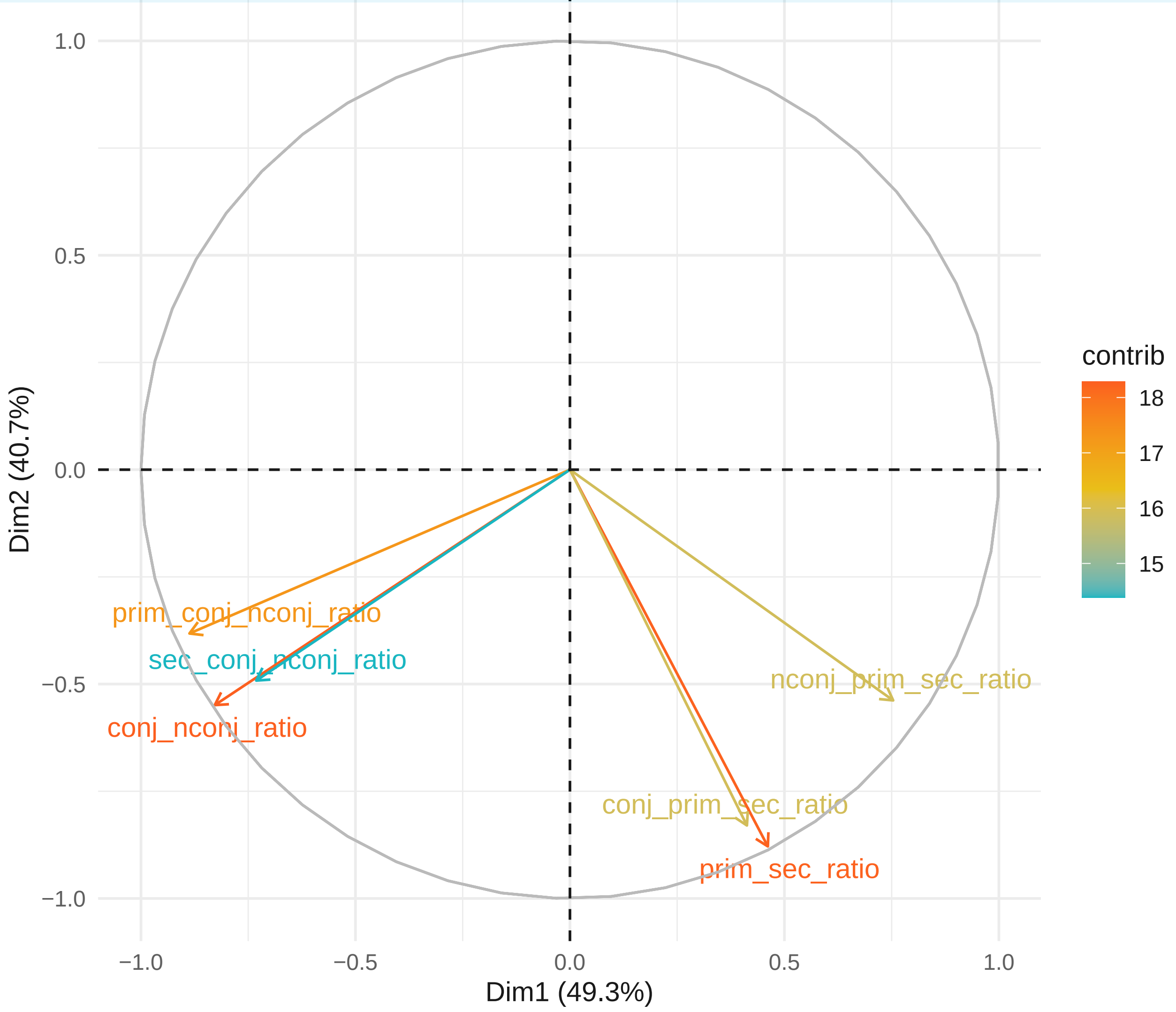
contrib
18
17
16
15
1.0
0.5
0.0
−0.5
−1.0
Dim2 (40.7%)
−1.0
−0.5
0.0
0.5
1.0
Dim1 (49.3%)
prim_conj_nconj_ratio
sec_conj_nconj_ratio
conj_nconj_ratio
nconj_prim_sec_ratio
conj_prim_sec_ratio
prim_sec_ratio
