# Supplementary material for: Interpreting Circulating Bile Acid Profiles in Pancreatic Cancer: The Role of Cholestasis and Its Management
Source: Cancers (Basel). 2026 Aug 2;18(15):2481. doi: 10.3390/cancers18152481 (PMC13463877; doi:10.3390/cancers18152481)
Supplement: Supplementary file 1 [file cancers-18-02481-s001.zip › Supplementary Figure S2.pdf]

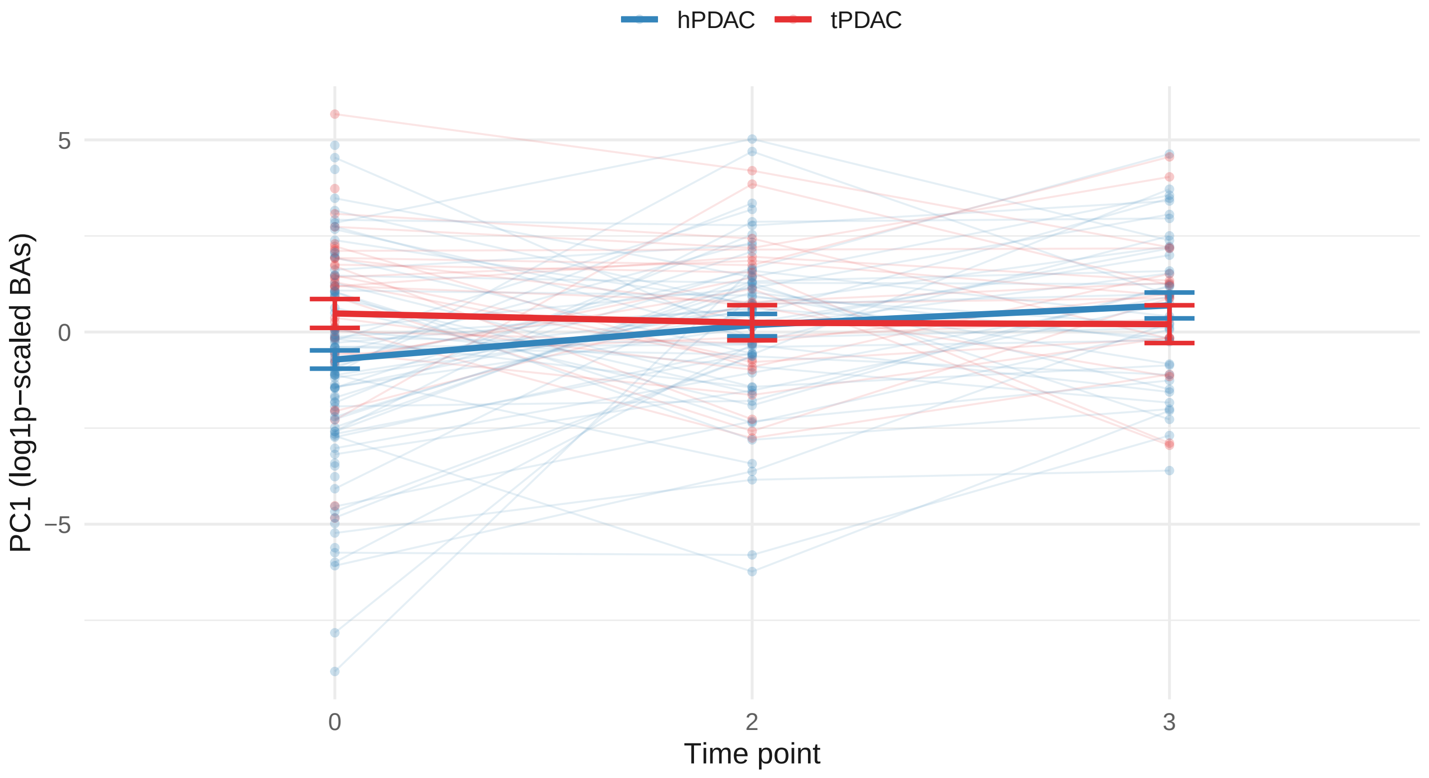
hPDAC
tPDAC
5
0
−5
PC1 (log1p−scaled BAs)
0
2
3
Time point
